# Changes in global translation elongation or initiation rates shape the proteome via the Kozak sequence

**DOI:** 10.1038/s41598-018-22330-9

**Published:** 2018-03-05

**Authors:** Julieta M. Acevedo, Bernhard Hoermann, Tilo Schlimbach, Aurelio A. Teleman

**Affiliations:** 1 German Cancer Research Center (DKFZ), 69120 Heidelberg, Germany; 2 Heidelberg University, 69120 Heidelberg, Germany; 3 National Center for Tumor Diseases (NCT), partner site, Heidelberg, Germany

## Abstract

The sequence context surrounding the AUG start codon of an open reading frame - the ‘Kozak sequence’ - affects the probability with which a scanning ribosome will recognize the start codon and start translating there. A significant number of transcripts in animals such as Drosophila contain weak Kozak sequences. This is predicted to cause constitutively low translation of these transcripts. We study here the additional possibility that these mRNAs have weak Kozak sequences to allow for the regulation of their translation in response to stress or altered cellular signaling. We find that transcripts with weak Kozak sequences are less sensitive to drops in global elongation rates and more sensitive to drops in global initiation rates compared to transcripts with strong Kozak sequences. This provides a mechanism by which changes in these global translation parameters differentially affect different pools of mRNAs depending on their Kozak sequence, thereby shaping the proteome. Interestingly, mRNAs with weak Kozak sequences are enriched for genes involved in neurobiology, suggesting that they constitute a functional group that can be translationally co-regulated.

## Introduction

Regulation of protein translation is a key step in the control of gene expression^1^. Protein translation provides an additional layer of regulation that is orthogonal to that of transcriptional regulation. Recent work has highlighted that translational mechanisms, analogously to transcriptional ones, differentially affect various subsets of mRNAs and thereby shape the proteome.

Translation rates have traditionally been thought to be regulated either globally, or in an mRNA specific manner. mRNA-specific regulation is achieved by the presence of structural or sequence motifs on the mRNA (e.g. uORFs, miRNA binding sites), whereas more global changes in translation are usually due to changes in initiation factor activity (e.g. eIF2α phosphorylation)^2,3^. Recent genome-wide studies, however, have shown that even ‘global’ mechanisms can differentially affect specific classes of mRNAs and thereby shape the proteome^4–6^. Which of these changes in global translation parameters lead to differential effects on subsets of mRNAs, and how this is achieved, are topics of current research. We discover here that changes in global elongation and global initiation rates differentially affect translation of different mRNAs, and that this effect is mediated by the Kozak sequence.

Cap-dependent translation involves recruitment of the pre-initiation complex (PIC) to the 5′ end of an mRNA followed by scanning to find an AUG initiation codon in an optimum sequence context. AUG recognition promotes scanning cessation, release of most initiation factors, and recruitment of the large ribosomal subunit to initiate elongation^7^. Efficient recognition of an initiation codon depends on its surrounding sequence. Pioneering studies by Marilyn Kozak identified the sequence CRCCaugG (R = purine, A or G) to be the optimal context for AUG recognition in eukaryotes^8–11^. Although a strong Kozak sequence is required for maximal translation of an mRNA, visual inspection of the genome of an animal such as Drosophila leads to the unexpected finding that many mRNAs have Kozak sequences that are predicted to be weak. Several interpretations of this finding are possible. One possibility is that these mRNAs encode for proteins that need to be expressed at constitutively low levels in cells. A second additional possibility, however, is that the Kozak sequences may also serve a regulatory role, with mRNAs containing strong or weak Kozak sequences forming distinct functional groups whose translation can be differentially regulated in a cell in response to changes in environment or cellular signaling. Here we explore this second explanation, and find that it is indeed the case. We find that when global elongation rates are reduced in a cell, mRNAs with weak Kozak sequences are relatively refractory, dropping less in their translation compared to mRNAs with strong Kozak sequences. Conversely, when initiation rates are reduced, mRNAs with weak Kozak sequences are more sensitive, dropping more in their translation compared to mRNAs with strong Kozak sequences. This interplay between global initiation and elongation rates via the Kozak sequence identifies how these ‘global’ regulatory mechanisms differentially affect different mRNAs, thereby shaping the proteome. This will likely be important for our understanding of how cells respond to stresses or environmental conditions that affect these global translation parameters.

## Results

### A significant number of transcripts contain weak Kozak sequences

**Figure 1:**
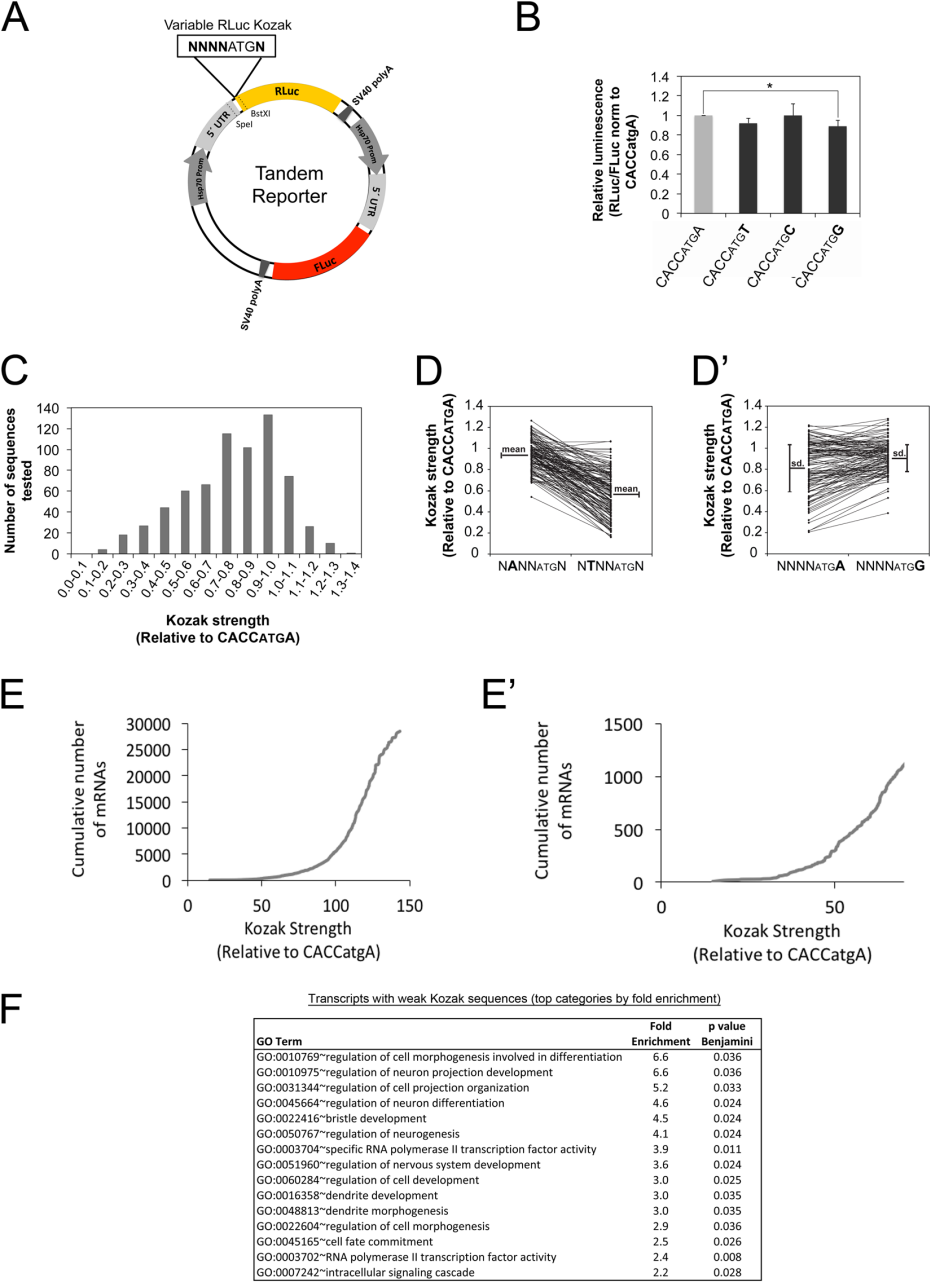
Transcripts with weak Kozak sequences form a functional group of mRNAs related to neurobiology (**A**) Schematic representation of the tandem RLuc-FLuc reporter used for high throughput analysis of Kozak qualities. The tandem reporter has both RLuc and FLuc open reading frames under the control of identical but independent promoters (Hsp70), 5′UTRs and SV40 polyA signals. The RLuc Kozak sequence can be exchanged easily via unique restriction sites. The screen was performed in biological triplicate in Drosophila Kc167 cells. (**B**) Changing the base at position +4 of RLuc in the context of a strong Kozak sequence in positions −4 to −1 has little effect on RLuc activity, indicating it likely does not impact enzymatic activity. Values are normalized to a co-transfected FLuc normalization control that is the same in all samples. Error bars: std. dev., n = 9 biological replicates, t-test *p < 0.05. (**C**) Histogram showing the frequency distribution of Kozak strengths obtained in the screen. (**D–D’**) Comparison of Kozak strengths for all sequences containing an A or T at position −3 (**D**) or an A or G at position +4 (**D**’). (**D**) As a general trend Kozaks with an A at -3 have better quality than Kozaks with T in position −3, seen as a difference in the means of the two distributions, (**D’**) In general, a G in +4 has a ‘dominant’ phenotype, causing an attenuation of the effects of positions −4 to −1, seen as a small deviation around the mean of the distribution compared to an A at +4. (**E–E’**) Correlation between Kozak usage in the Drosophila genome and Kozak quality. The fly genome is enriched in strong Kozak sequences, nonetheless, 112 transcripts in total have weak Kozak sequences (<40% of the canonical CACCatgA). A zoom on Kozak sequences with strengths <70% is shown in panel E’. A scatter plot showing usage vs. strength for all Kozak sequences is in Supplementary Figure S2B. (**F**) Gene Ontology analysis on weak-Kozak containing transcripts identifies an enrichment for functional groups involved in neuron biology and transcriptional regulation. Enrichment analysis was performed using DAVID (version 6.7)^14^ on the 688 transcripts with Kozak qualities below 70% of the strong Kozak (CACCatgA). GO categories are shown here sorted by fold-enrichment, with a maximum cut-off for Benjamini-corrected p values of 0.05. For p-value sorted terms, see Supplementary Figure S2C.

While studying upstream Open Reading Frames in Drosophila^12^, we noticed that a number of transcripts contain Kozak sequences on their main ORFs that do not match the optimal consensus CRCCAUGG (R = purine, A or G)^8,9^. One option is that the Kozak sequences on these transcripts are indeed weak, as predicted by their nucleotide sequence. An alternate option is that these Kozak sequences actually do support efficient translation in Drosophila despite not matching the mammalian optimal consensus, since the Kozak sequence has not been studied in depth in Drosophila. To systematically analyze Kozak sequence quality in Drosophila, we performed a high throughput mutagenesis study and measured the Kozak quality of a large number of different sequences in Drosophila Kc167 cells. To this end we designed a tandem reporter plasmid containing Renilla Luciferase (RLuc) and Firefly Luciferase (FLuc) with identical but independent promoters (Hsp70), 5′UTRs and SV40 polyA signals (Fig. 1A). Whereas RLuc serves as the experimental reporter, FLuc served as a normalization control. This tandem setup ensures equal stoichiometry of the two reporters, significantly improving inter-experimental variability compared to co-transfection. Comparison of the tandem reporter bearing a strong Kozak sequence (CACCatgA) versus the reporter bearing a weak Kozak sequence (TTTTatgA) yielded the expected difference in normalized RLuc luciferase counts (Supplementary Figure S1A). To exclude that the difference in luciferase counts may be due to effects of the Kozak sequence on mRNA stability, we quantified both luciferase activity and mRNA levels of the reporters. The ratio, indicating the amount of protein synthesized per amount of mRNA, which we will call here ‘translation efficiency’, also showed the expected drop (Supplementary Figure S1A’). We next performed site-directed mutagenesis of the RLuc Kozak sequence using oligos with random bases at positions −1 to −4 and +4. Since the base at position +4 codes for the 2^nd^ amino acid of RLuc, we first tested if changing nucleotide +4 has a significant impact on RLuc enzymatic activity. In the context of an optimal Kozak sequence at positions −1 to −4 (CACC) which ensures efficient translation, changing the base at +4 had little impact on RLuc activity (Fig. 1B).

We generated and tested 680 different Kozak constructs thus covering 66% of all possible Kozak sequences. These measurements will likely serve as a useful resource for labs working on translation in Drosophila (Supplementary Table S1). A histogram of Kozak strength (RLuc/FLuc normalized to the strength of CACCatgA) for all tested sequences is shown in Fig. 1C. There is a 10-fold dynamic range in the ability of different sequences to promote translation initiation, but most sequences support initiation at rates similar to those of the ‘canonical’ CACCatgA sequence (Fig. 1C). To better understand the logic underlying Kozak strength, we next analyzed the contribution of each individual position to the strength of the combined Kozak sequence. To this end, we plotted Kozak strengths for all sequences containing a particular nucleotide in a specific position. For instance, Fig. 1D shows the strengths of all Kozak sequences containing an A at position −3, compared to a T at position −3, with sequence pairs differing only in this position connected by lines. This type of analysis revealed two general trends. First, as can be seen in Fig. 1D, some nucleotides generally lead to a stronger Kozak sequence compared to others. For instance, sequences with an A at position −3 are generally stronger than the equivalent sequences with a T at position −3 (Fig. 1D). This can be quantified by calculating the mean of the distribution of Kozak strengths for each nucleotide at each position (Fig. 1D, mean for A at −3 = 0.94, mean for T at −3 = 0.57). Second, some nucleotide/position combinations have a more dominant effect on Kozak strength than others. For instance, by comparing all sequences with an A at position +4 versus a G at position +4 (Fig. 1D’) one sees that the means of the two distributions are not very different, but a G at +4 causes the sequence strengths to cluster more tightly around the mean compared to the A at +4. This leads to the non-linear effect that in some cases changing the A at +4 to a G causes the Kozak strength to increase, whereas in other cases it causes the Kozak strength to decrease (Fig. 1D’). This can be quantified by measuring the standard deviation in the distribution of Kozak strengths for any nucleotide at any position (0.25 for G at +4, and 0.16 for T at +4). The resulting means and standard deviations for each nucleotide at each position are shown in Supplementary Table S2. Using these values, we calculated the Kozak quality for all Kozak sequences (see Materials & Methods for details) yielding a very good correlation between calculated and measured Kozak quality (Supplementary Figure S2A). The predicted Kozak strength for all possible Kozak sequences is provided in Supplementary Table S3, and for all Drosophila transcripts in Supplementary Table S4. The strongest Kozak sequence we identified is AAAAatgG, which is similar to the most frequent Kozak sequence transcriptome-wide in Drosophila, which is CAAAatgG.

Using this comprehensive database of Drosophila Kozak quality, we analyzed the presence of each Kozak sequence in the fly transcriptome, as annotated by Flybase^13^. Generally, transcripts with strong Kozak sequences are more numerous than transcripts with weak Kozak sequences (Fig. 1E and Supplementary Figure S2B). That said, many strong Kozak sequences are not abundant in the transcriptome (e.g. the sequence AAGGatgG has a strength of 132% compared to CACCatgA, but is present only 37 times in the transcriptome) (Supplementary Figure S2B). Furthermore, a good number of weak Kozak sequences are present in the genome, with >1000 transcripts containing Kozak sequences with strengths less than 70% that of the CACCatgA consensus, and 112 transcripts containing Kozak sequences with strengths less than 40% that of the CACCatgA consensus (Fig. 1E’). For instance, all 9 transcript isoforms of the Lkb1 gene contain GTTTatgC as a Kozak sequence, which we measured to support translation at only 16% the level of the CACCatgA consensus. Hence, the frequency of a Kozak sequence in the genome does not appear to be a good proxy for Kozak ‘quality’ or strength, since Kozak frequency and strength do not correlate very well.

To test if mRNAs with weak Kozak sequences consist of a group of functionally related genes, we performed a Gene Ontology enrichment analysis using the DAVID v6.7 server^14^ on the 688 transcripts with Kozak strengths below 70% of the consensus Kozak (CACCatgA). This revealed that transcripts with weak Kozaks are enriched for genes involved in neuron biology and in transcriptional regulation (Fig. 1F sorted by enrichment, and Supplementary Figure S2C sorted by p-value).

### Transcripts with weak Kozak sequences are less sensitive to global drops in translation elongation rates

**Figure 2:**
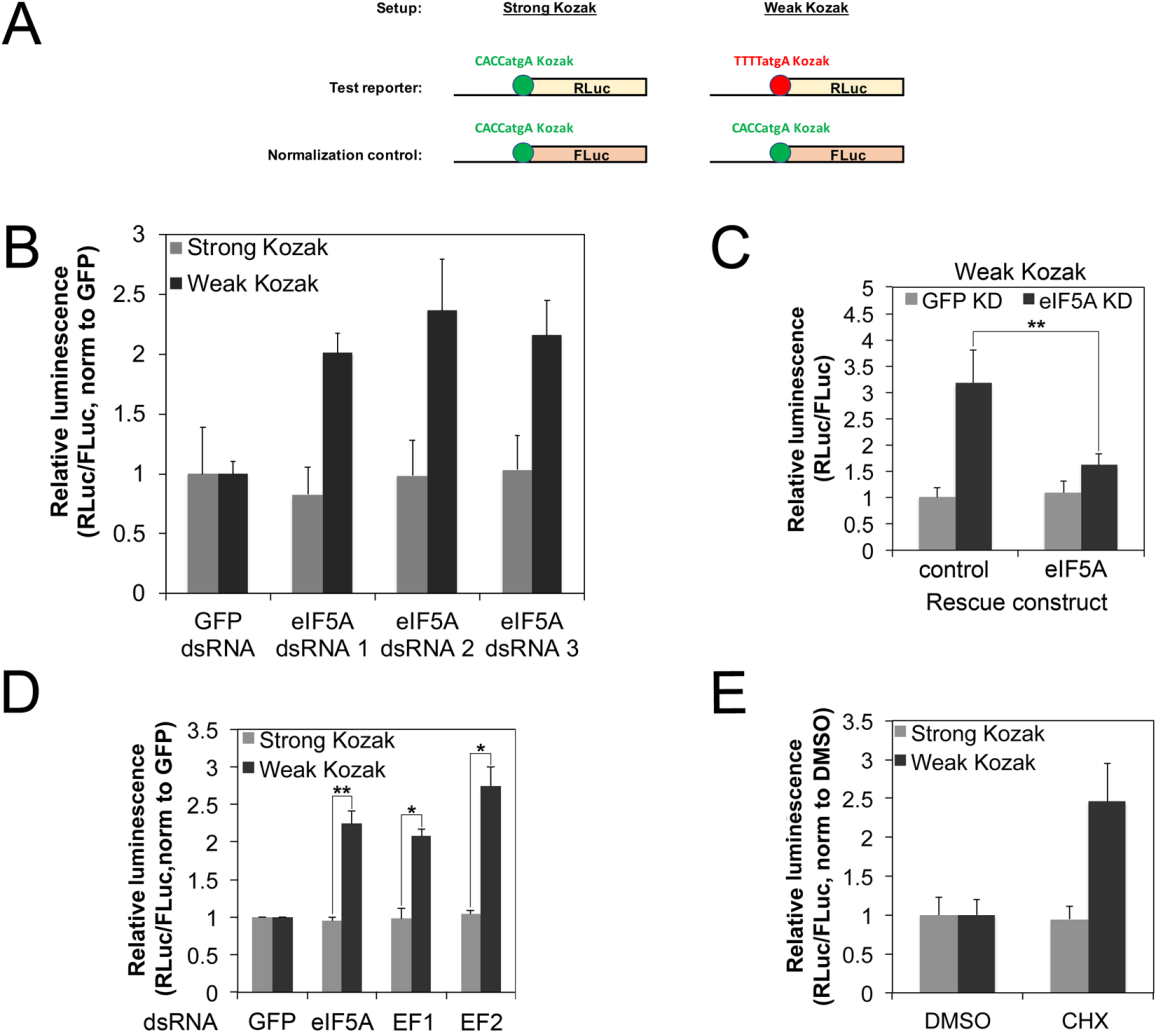
Transcripts with weak Kozak sequences are spared when global elongation rates drop (**A**) Schematic diagram of the assay setup. Test Renilla Luciferase (RLuc) reporters carrying either a strong or a weak Kozak sequence were co-transfected together with a Firefly Luciferase (FLuc) normalization control carrying a strong Kozak sequence. All graphs in this figure show the RLuc/FLuc ratio, which normalizes out global changes in translation rates as well as technical artefacts. (**B**) eIF5A knockdown differentially affects translation of transcripts bearing strong or weak Kozak sequences. S2 cells were treated with two independent dsRNA targeting eIF5A or GFP dsRNA as a control, and then transfected with strong or weak Kozak reporters as shown in (**A**). The RLuc/Fluc ratio obtained for every dsRNA treatment was normalized to that of the GFP control. Error bars: std. dev., n = 4 biological replicates. (**C**) The eIF5A knockdown phenotype can be rescued by reconstituting eIF5A expression via an RNAi-resistant expression construct, indicating it is specific. Kc167 cells treated with dsRNA targeting the eIF5A 3′UTR or GFP (negative control) were transfected with the weak Kozak reporter together with a construct expressing either GFP (negative control) or the eIF5A open reading frame. Error bars: std. dev., n = 4 biological replicates, **p < 0.01 (**D**,**E**) Constructs with weak Kozak sequences are relatively spared compared to strong Kozak sequences when elongation rates are reduced. Elongation rates were reduced either by (**D**) knocking down elongation factors 1 or 2 (EF1 or EF2), or eIF5a, or (**E**) by treating cells with low levels of cycloheximide (CHX, 0.25 ug/ul). RLuc/FLuc ratios for each construct were normalized to the respective negative control treatments (GFP dsRNA for D, DMSO vehicle for E). Error bars: std. dev. n = 4 biological replicates, *p < 0.05, **p < 0.01 by t-test.

We next asked whether any cellular factors or environmental inputs differentially affect translation of this class of transcripts containing weak Kozak sequences, compared to transcripts with strong Kozak sequences. To this end, we performed a targeted RNAi screen, looking for factors that differentially affect translation of a luciferase reporter bearing a weak Kozak sequence compared to a reporter with a strong Kozak (Supplementary Figure S3A). We screened a subset of translation initiation factors (eIFs), as eIFs are the best candidates to directly modulate the start codon selection process. We individually knocked down the expression of selected eIFs in S2 or Kc167 cells and then transfected the tandem reporter with RLuc bearing either a strong (CACCatgA) or a weak (TTTTatgA) Kozak (Supplementary Figure S3A). We normalized all data to a co-transfected FLuc control reporter, in order to normalize out changes in global translation rates, and scaled the values so that the GFP control knockdowns are set to 1. Since this is a screen, we did not assess knockdown efficiency for each individual dsRNA, hence we cannot exclude false-negatives. Nonetheless, the screen was successful, as it identified eIF5A as a factor that differentially affects translation of the strong versus the weak Kozak-containing reporters (Supplementary Figure S3B–B’) in a consistent manner in both cell lines (Supplementary Figure S3B”). eIF5A knockdown causes a drop in translation of both strong and weak-Kozak containing transcripts, but it less strongly blunts translation of weak-Kozak containing transcripts (RLuc counts in Supplementary Figure S3C). This can be observed as a relative increase in expression of the weak-Kozak containing reporter when normalized to the strong-Kozak normalization control (Supplementary Figure S3B–B’). Using two independent dsRNA in addition to the one in the screen, we confirmed that specific knockdown of eIF5A blunts translation of a weak-Kozak containing transcript less strongly than translation of a strong-Kozak containing transcript (Fig. 2A,B). We also confirmed that restoring expression of eIF5A in eIF5A knockdown cells via a non-targeted eIF5A cDNA leads to a significant rescue of the observed phenotype (Fig. 2C).

Although eIF5A was initially characterized as an initiation factor, its function was later linked mainly to translation elongation^15–17^. We therefore asked if the effect of eIF5A on Kozak-dependent translation is due to its role as an elongation factor, by knocking down other elongation factors and testing if they recapitulate this phenotype. Interestingly, knockdown of EF1 or EF2 showed the same effect as eIF5A knockdown, leading to an increase in the relative expression of the weak-Kozak RLuc reporter compared to the strong-Kozak normalization FLuc control (Fig. 2D). To confirm this observation pharmacologically, we transfected cells with the Kozak tandem reporters and treated them with low levels of the elongation inhibitor cycloheximide (CHX) which partially inhibit translation (Supplementary Figure S3D) in agreement with previous reports^18^. In agreement with the knockdown experiments, pharmacological inhibition of elongation also led to enhanced relative expression of the weak Kozak containing tandem reporter (Fig. 2E). In sum, these results indicate that global drops in elongation rates in a cell lead to differential effects on the translation of transcripts bearing strong versus weak Kozak sequences. Compared to transcripts with strong Kozak sequences, transcripts with weak Kozak sequences are comparatively resistant to drops in elongation rates. One rationalization is that when initiation rates on a transcript are rate-limiting due to presence of a weak Kozak sequence, elongation rates are not limiting, and hence can drop without having a significant impact. This mechanism thereby causes a ‘global’ change in translation rates to have differential effects on different classes of mRNAs, thereby shaping the proteome.

### mRNAs with weak Kozak sequences are relatively insensitive to changes in mRNA-specific elongation rates

**Figure 3:**
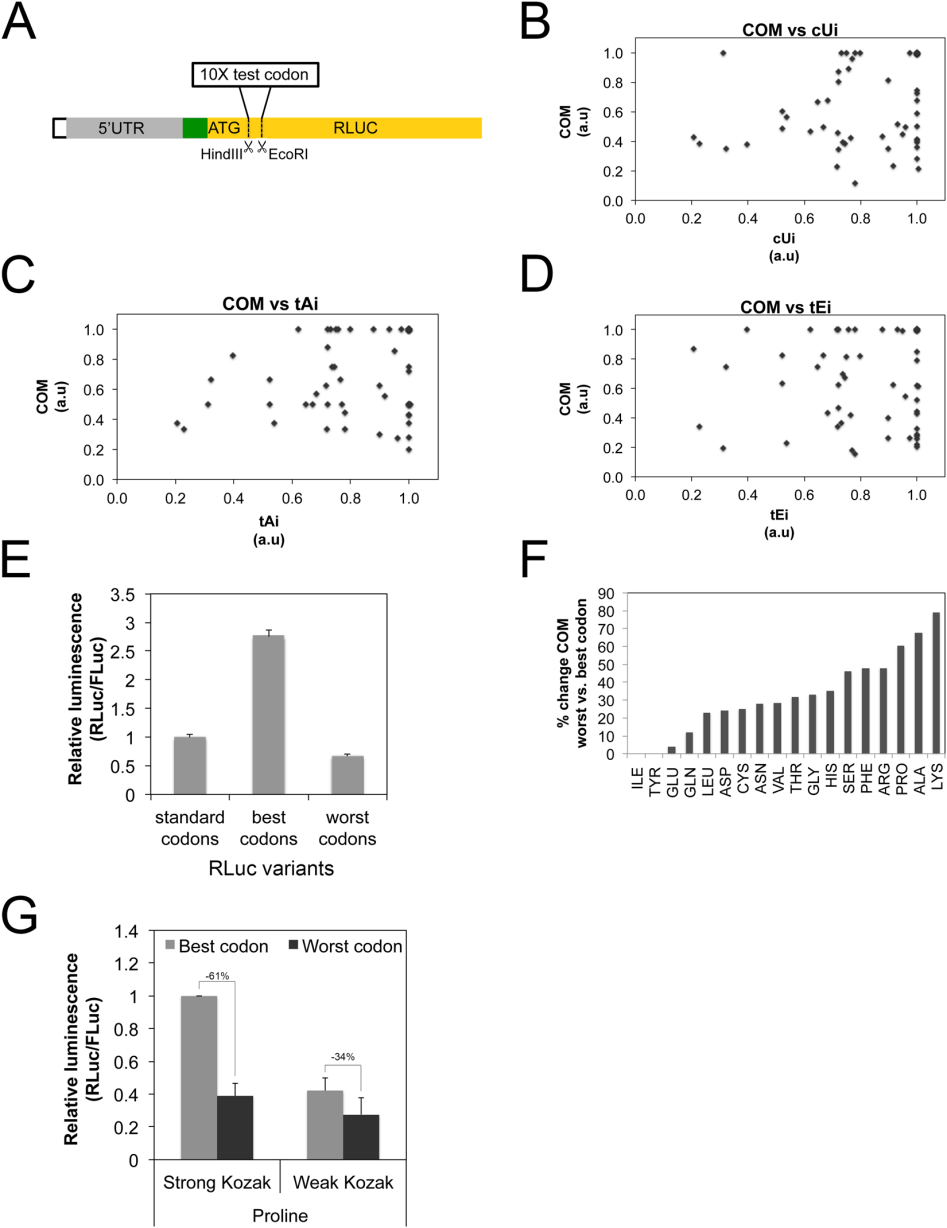
Transcripts with weak Kozak sequences are spared when mRNA-specific elongation rates drop. (**A**–**E**) Experimentally measured codon qualities do not correlate with frequently used, computational codon quality scores. (**A**) Schematic representation of the reporter used to measure codon qualities, yielding Codon Optimality Measurements (COM) for all codons. The tandem reporter containing a strong Kozak sequence (green box) was modified to introduce, restriction sites immediately after the RLuc ATG to introduce ten tandem copies of a codon to be tested. RLuc activity was first normalized to the FLuc normalization control, and then within each group of codons coding for one amino acid, COM measurements were normalized so that the strongest codon has a value of 1. Done in 6 biological replicates. (**B**–**D**) Codon Optimality Measurements (COM) do not correlate with frequently used, computationally derived, codon quality scores such as (**B**) the codon usage index (cUi), (**C**) the tRNA adaptaion index (tAi) or (**D**) the translation efficiency index (tEi). (**E**) RLuc expression levels are affected as expected following codon improvement or worsening based on COM scores. RLuc versions with the best and the worst possible codons were synthetized and RLuc values were normalized to a co-transfected FLuc normalization control. n = 7 biological replicates. (**F**) Percent of COM change between the best and the worst codon for each amino acid shows that codon selection for some amino acids (eg Lysine) has the potential to impact translation more than codon selection for others (e.g. Isoleucine). (**G**) A slow elongation rate, due to the presence of sub- optimal codons following the ATG, causes a larger drop in translation of transcripts bearing strong Kozak sequences compared to those with weak Kozak sequences. Reporters contain either a strong or a weak Kozak, followed by 10 tandem copies of either the best or the worst Proline codons Error Bars: std. dev. n = 4 biological replicates.

The interaction presented above takes place between the global cellular elongation rate and the Kozak sequence on individual mRNAs. Elongation rates, however, also vary between individual mRNAs within a cell depending on the codon usage of the Open Reading Frame^19^. While certain codons for a given amino acid are translated rapidly, others are not^20,21^. The rate at which each codon is translated is thought to depend on the supply versus the demand for charged tRNAs: i.e. the expression level of a tRNA and how often the corresponding codon is present in the transcriptome^22^. To test the concept presented above, that changes in elongation rates differentially affect transcripts with strong versus weak Kozak sequences, we asked whether we can also observe this differential effect if we modulate elongation rates in an mRNA specific way. To test this, we changed transcript-specific elongation rates by changing codon usage on the transcript. We first measured the codon quality for all codons in Drosophila to identify codons that are translated efficiently or poorly. Until now, several indexes that estimate codon translation efficiency have been calculated^22^, and the impact of the 5′UTR on protein translation has been systematically analyzed in yeast^23^, however to our knowledge no systematic experimental measurement of codon strength in animals has been performed. We designed a tandem RLuc-FLuc reporter that allowed us to perform codon optimality measurements (Fig. 3A). This reporter contains restriction sites in the RLuc ORF, directly downstream of the ATG, into which we cloned ten tandem copies of the codon to be tested. This leads to production of RLuc containing 10 copies of an amino acid at its N-terminus. Since the presence of ten copies of a given amino acid can affect activity and stability of the RLuc protein, comparisons between different amino acids are not possible. However, for any one amino acid, the relative translation rate of the different codons coding for that amino acid can be compared. Hence we normalized the Codon Optimality Measurements (COM) so that the best codon for any individual amino acid is set to 1, and the quality of all other codons coding for that same amino acid are calculated relative to that. Results of our Codon Optimality Measurements (COM) are presented in Supplementary Table S5. Interestingly, these experimentally-derived codon optimality measurements do not correlate well with frequently-used computational predictions for codon quality, such as codon usage (Fig. 3B), translation adaptation index (Fig. 3C) and the translation efficiency index, calculated for Drosophila following^22^ (Fig. 3D), suggesting that codon quality needs to be measured for the system of interest. (Note that to be able to directly compare our COM to these other indexes, we normalized the values of these indexes within each amino acid, so that the best codon for each amino acid has a strength value of 1.) As a validation of our Codon Optimality Measurements, we synthesized RLuc with the best or the worst codons according to our COM results. As expected, the RLuc bearing the best codons is expressed more efficiently than that bearing the worst codons (Fig. 3E). To discard possible effects of the codon changes on mRNA stability we directly measured translational efficiency of the reporters bearing the best and worst codons by quantifying both luciferase activity and mRNA levels and calculated translation efficiency as the ratio of the two. The worst codon containing RLuc had a lower translation efficiency than the best codon containing RLuc (Supplementary Figure S4A). Although this does not exclude the possibility that some of the Codon Optimality Measurements may be due in part to effects on mRNA stability, this indicates that on average across all codons this is not the case. Furthermore, the 10 tandem “AAA” and “UUU” codons might reduce luciferase levels by inducing frameshifting. Nonetheless, our compilation of Codon Optimality Measurements (Supplementary Table S5) will likely be useful in the future for optimizing or manipulating gene expression in Drosophila. Analysis of these data reveals that some amino acids show a large difference in translation efficiency between the best and the worst codons for that amino acid (e.g. alanine or lysine, Fig. 3F) whereas some amino acids show little to no difference between codons (e.g. isoleucine, Fig. 3F). Hence the magnitude of the effect on translation that is possible as a consequence of codon choice depends on the identity of the amino acid.

Using these experimentally derived Codon Optimality Measurements, we tested the hypothesis that mRNAs with weak Kozaks are less sensitive to drops in elongation rates compared to mRNAs with strong Kozaks. To this end we selected one of the amino acids whose best and worst codons showed significant differences in translation rates: Proline (Fig. 3F). We generated reporters containing either a strong or a weak Kozak, followed by the ATG of RLuc, followed by 10 tandem repeats of the best or the worst proline codon. We confirmed that the drop in RLuc activity caused by the presence of the worst Pro codons was indeed due to a translation effect, and not an mRNA effect, by measuring mRNA levels and calculating the translation efficiency (Supplementary Figure S4B). We then compared the magnitude of the translation drop caused by the poor-codon elongation slowdown for the reporter with a strong Kozak versus the weak Kozak (Fig. 3G). As expected, slowing down elongation on the mRNA with a weak Kozak had less of an effect than the same elongation slowdown on the mRNA with a strong Kozak. The reporter bearing 10 prolines with a strong Kozak sequence drops in translation by 61% when poor codons are replaced for the good, whereas it only drops by 34% if the Kozak sequence is weak (Fig. 3G). In sum, these data indicate that changes in cellular parameters that affect codon quality (e.g. changes in tRNA expression levels) will differentially affect mRNA translation depending on whether the Kozak sequence is weak or strong. mRNAs with weak Kozak sequences are preferentially spared either when global elongation rates drop, or when elongation rates on a specific mRNA drop due to its codon usage.

### mRNAs with weak Kozak sequences are highly sensitive to drops in initiation rates

**Figure 4:**
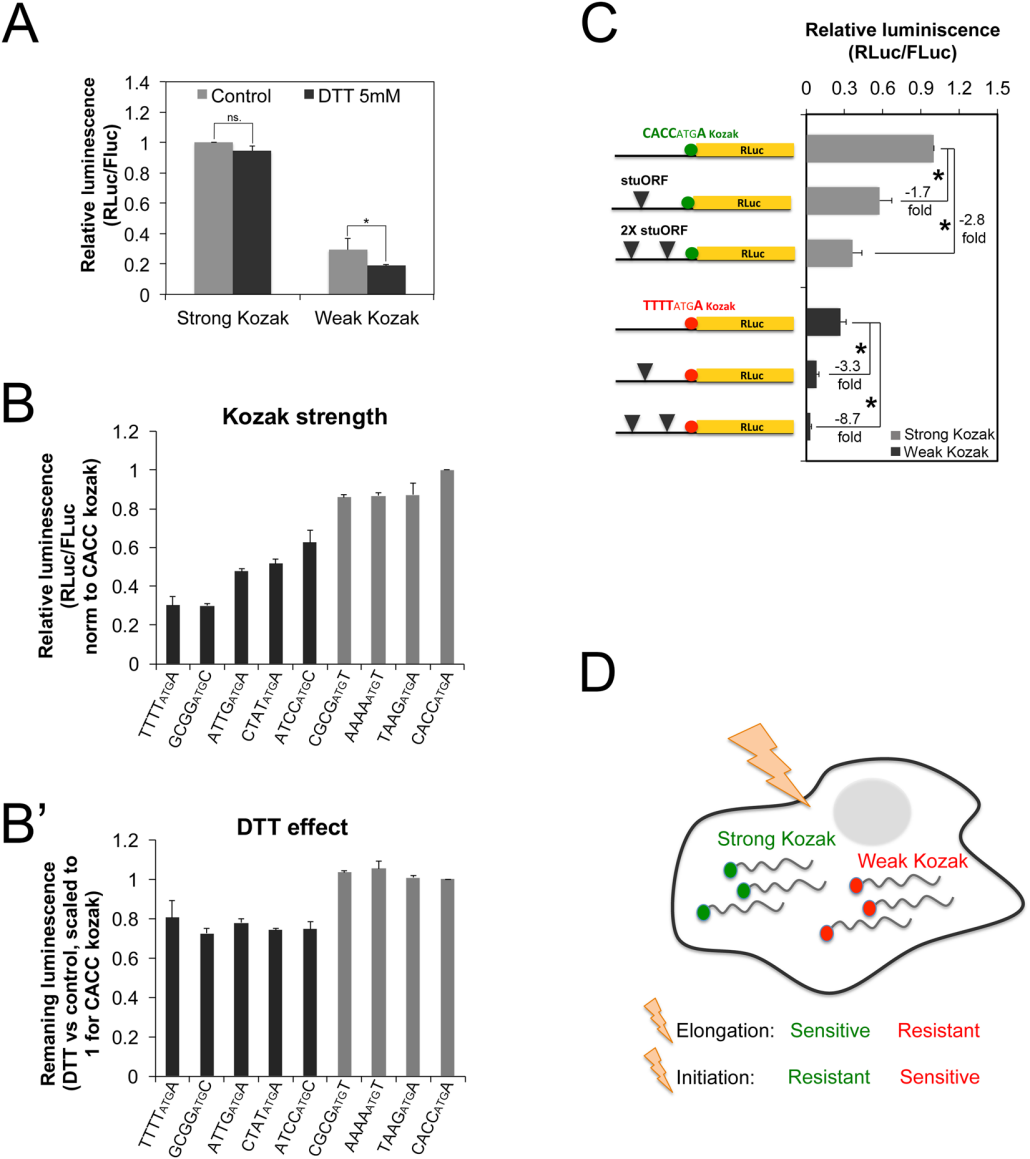
Transcripts with weak Kozak sequences are particularly sensitive to global or mRNA-specific drops in initiation rates. (**A**–**B**’) Transcripts with weak Kozak sequences are more sensitive to reductions in global translation initiation rates due to treatment of cells with DTT, compared to transcripts with strong Kozak sequences. Kc167 cells transfected with indicated reporters, treated +/− DTT (5 mM, 12 h). (**A**) Translation of RLuc transcripts bearing either a strong or a weak Kozak sequence. RLuc values are normalized to an FLuc normalization control reporter, and scaled relative to the strong Kozak reporter in the untreated condition. (**B**) A range of transcripts with Kozak sequences of varying strengths (**B**) is tested for the inhibitory effect of DTT (**B**’). In (**B**), RLuc values are normalized to an FLuc control reporter, and scaled relative to the CACCatgA reporter. In (**B**’), RLuc values are normalized to an FLuc control reporter, then the ratio +DTT/−DTT is calculated, and this is scaled to 1 for the CACCatgA reporter. n ≥ 6 biological replicates. *p < 0.05 by t-test. (**C**) Compared to transcripts with strong Kozak sequences, transcripts with weak Kozak sequences are more sensitive to reductions in mRNA-specific translation initiation rates due to the presence of ‘strong Kozak containing upstream Open Reading Frames’ (stuORFs)12. RLuc reporters bearing either one or two uORFs in the 5′UTR of strong or weak Kozak containing reporters in Kc167 cells, normalized to an FLuc control reporter. Error bars: std. dev. n = 8 biological replicates. (**D**) Schematic representation of the two classes of mRNAs - those with weak and those with strong Kozak sequences - being differentially sensitive to drops in either elongation or initiation rates.

The results presented thus far suggest that mRNAs with weak Kozak sequences are spared when elongation rates drop, because initiation rates, and not elongation rates, are limiting on these mRNAs. Following this logic, mRNAs with weak Kozak sequences should be very sensitive to drops in initiation rates, since initiation rates are limiting on these mRNAs. To test this, we reduced initiation rates in two different ways: globally using DTT, and mRNA-specifically using upstream Open Reading Frames (uORFs). DTT induces ER stress, leading to phosphorylation of eIF2α, thereby reducing global initiation rates^24^. As expected, DTT caused a stronger drop in translation of an mRNA containing a weak Kozak sequence compared to an mRNA containing a strong Kozak (Fig. 4A). In order to test a range of Kozak strengths spanning the difference between the “strong” and the “weak” Kozak, we selected a panel of reporters from our screen (Fig. 1). Consistent with the data presented above, reporters with weaker Kozak sequences (Fig. 4B) showed a stronger drop in expression upon treatment with DTT compared to reporters with stronger Kozak sequences (Fig. 4B’). Interestingly, there seems to be a bimodal response to DTT with a strength threshold below which Kozak sequences respond more dramatically to the DTT treatment (categorized in grey or black, Fig. 4B–B’). This threshold may represent the Kozak strength below which initiation becomes limiting. To reduce initiation rates in an mRNA-specific manner, we introduced upstream Open Reading Frames containing strong Kozak sequences (stuORFs)^12^, which are translated by ribosomes, and thereby reduce the rate of initiation on the main ORF. As shown in Fig. 4C, the presence of one or two stuORFs on a transcript containing a strong Kozak sequence (green) caused translation to drop by 1.7- and 2.8-fold respectively. In contrast, introduction of the same stuORFs into the transcript containing a weak Kozak sequence (red) caused translation to drop more dramatically, by 3.3- and 8.7-fold respectively (Fig. 4C).

In sum, the data presented here indicate that mRNAs in a cell can be broadly categorized into two groups - those with strong Kozak sequences versus those with weak Kozak sequences (Fig. 4D). Compared to the strong Kozak containing transcripts, the weak Kozak containing transcripts are resistant to drops in elongation but sensitive to drops in initiation rates. This is the case both for global changes in elongation and initiation rates, as well as mRNA-specific changes. This provides a mechanism by which global changes in elongation or translation rates differentially impact translation of various mRNAs in a cell.

## Discussion

Protein translation is a complex and tightly regulated process that is controlled both globally and at an mRNA specific level^2^. We discover here a mechanism by which global changes in elongation or initiation rates differentially affect two groups of mRNAs - those with strong Kozak sequences and those with weak Kozak sequences. While strong Kozak containing mRNAs are very sensitive to drops in global elongation rates, mRNAs with weak Kozaks are more resistant. This is likely because initiation, and not elongation, is limiting on transcripts with weak Kozak sequences, hence elongation rates do not impact translation of this class of mRNAs very much. Thus any physiological condition inducing a decrease in mRNA elongation rates may differentially affect translation of these classes of mRNAs, thereby shaping the proteome. Regulation of protein translation at the elongation level is starting to be considered an important step in the control of gene expression^25–27^. One condition that selectively reduces translation elongation rates is the stimulation of neurons with Glutamate or NMDA, leading to activation of the NMDA receptor and an increase in inhibitory phosphorylation of EF2^28,29^. Intriguingly, we find that transcripts with weak Kozak sequences are enriched for neuron-related genes (Fig. 1F), suggesting that such mechanisms may indeed be important in neurons. Likewise, we find that changes in mRNA-specific elongation rates due to codon usage also differentially affect mRNAs with weak versus strong Kozaks (Fig. 3G). Physiological conditions or stresses that lead to altered tRNA representation will presumably alter codon optimality, thereby changing elongation rates on mRNAs. For instance, changes in tRNA expression levels have been reported to occur in mammalian cells when they switch from proliferation to quiescence^30^. Unfortunately, we could not find a condition that selectively decreases elongation but not initiation rates in our Drosophila cells in culture, hence it will be interesting to apply our findings to other model systems in the future. Interestingly, a recent study found that in response to stress, Saccharomyces cerevisiae produces ribosomes lacking Rps26. Since Rps26 normally helps ribosomes recognize strong Kozak sequences, this leads to a drop in translation of transcripts with strong Kozak sequences while increasing translation of transcripts with weak Kozak sequences^31^. Hence this mechanism and the mechanism we describe here might work in parallel in response to stress to tilt the balance towards translation of transcripts with weak Kozak sequences.

The data we present here can also be viewed from a different perspective - that of the Kozak sequence. Recent studies have analyzed the impact of the Kozak sequence on translation rates on an ‘omic’ scale, and unexpectedly found only weak correlations^32,33^. Our data show that the impact of the Kozak sequence on translation depends on the relative rates of elongation and initiation globally in a cell and specifically on a transcript. When elongation rates are low and limiting, the presence of a strong versus a weak Kozak makes little difference on translation of that transcript. For instance, when analyzing the data presented in Fig. 3G from the perspective of the Kozak sequence, one sees that swapping a strong Kozak for a weak one makes a big difference if the open reading frame contains optimal codons (bars 3 versus 1, Fig. 3G) whereas it makes little difference if the open reading frame has poor codons (bars 4 versus 2, Fig. 3G). Likewise, initiation rates modulate the impact of the Kozak sequence on translation. When initiation rates are high and not limiting, the Kozak sequence has a mild impact on mRNA translation. For instance, in Fig. 4C in the absence of stuORFs, replacing the strong Kozak sequence with a weak one causes a 4-fold drop in translation (1st vs 4th bars). In contrast, when initiation rates become limiting, the Kozak sequence has a strong impact on translation rates. In Fig. 4C, replacing the strong Kozak sequence with a weak one in the presence of 1 stuORF causes a 7-fold drop in translation, and in the presence of 2 stuORFs it causes a 12-fold drop in translation. In summary the impact of the Kozak sequence on translation depends on whether initiation or elongation rates are limiting on an mRNA, due to both global and mRNA-specific influences (Supplementary Figure S5A). This likely convolutes the impact of the Kozak on translation on an ‘omic’ scale, leading to the poor correlations seen by others.

In this report, we measured the quality of codons using a luciferase reporter assay. We confirmed the validity of these measurements by synthesizing versions of renilla luciferase (RLuc) containing either good or bad codons, and found the RLuc protein levels to change as expected. Various indexes have been calculated previously to predict codon optimality: codon usage (cUi), tRNA adaptation index (tAi) and the translation efficiency index (tEi)^22^. Among these indexes the tEi is the most comprehensive because it considers the tRNA supply and demand. tRNA supply, however, is only deduced from the tRNA gene number, ignoring possible effects of tRNA gene transcription or tRNA modifications. Unexpectedly, we find that none of these computational predictions match well with our codon quality measurements. This suggests that these various computational indexes for codon quality should be used with caution.

In sum, our work identifies two classes of mRNAs in cells - those with strong Kozak sequences and those with weak Kozak sequences - which are differentially regulated in response to global changes in elongation and initiation rates.

## Materials and Methods

### Generation of reporter constructs

A plasmid containing the Hsp70 basal promoter and the CG43674 5’UTR controlling the expression of the Renilla luciferase ORF (RLuc) followed by a SV40 poly-adenylation signal was previously generated in our laboratory (pSS177)^12^. The same 5′UTR was amplified by PCR using PstI and NcoI containing oligos and was cloned into a plasmid containing the firefly luciferase ORF (FLuc), with the same hsp70 basal promoter and the same SV40 poly-adenylation signal as pSS177 (pJA24). The following mutations in both plasmids were performed before combining them to generate a tandem RLuc-FLuc reporter. A BstX1 site was introduced in the RLuc sequence by performing a G to A silent mutation at position 1202 and a SpeI restriction site was removed in the 5′UTR of the pJA24 plasmid by mutating two bases of the target sequence (ACTAGT was changed to GGTAGT). The new pSS177 and pJA24 mutated versions were digested with BamHI/SalI and BglII/SalI/PdmI respectively and the products of the digestion with compatible ends were ligated to generate a tandem reporter. The resulting tandem vector expresses the two reporter genes under the control of independent but identical basal promoters, 5′UTR and SV40 poly-adenylation signals. The RLuc Kozak sequence can be easily exchanged by replacing the region encompassed between the unique SpeI and BstX1 restriction sites.

For generating the Codon Optimality Measurement reporter a HindIII and an EcoRI site were introduced immediately after the ATG of the RLuc in the tandem reporter. Runs of 10x codons were introduced in this reporter by oligo cloning using the above mentioned restriction sites. All possible codons were tested except: CCC and GGG for DNA synthesis technical reasons and TGG and ATG since they are the only codons coding for Tryptophan and Methionine respectively.

To generate the uORF + kozak tandem reporters two plasmids that are identical to pSS177 but contain either 1 or 2 stuORF in the CG43674 5′UTR were used as starting point. Mutations in the Kozak sequence of these reporters were performed before combining them with the FLuc reporter (pJA24).

### Kozak high-throughput mutagenesis

For performing Kozak high-throughput mutagenesis, oligos with random bases at the Kozak positions −4 to −1 and +4 were used. The pool of fragments with mutated Kozaks of unknown identity was ligated into the Tandem RLuc-FLuc reporter. Colonies were picked and processed using the Nucleospin96 Plasmid kit (Macherey-Nagel). The identity of each of the purified plasmids was determined by sequencing.

### Synthesis of RLuc variants

RLuc variants with the best or worst codons according to our codon optimality measurements were synthetized using the GeneWiz gene synthesis service. For those amino acids showing no codon preference one codon was arbitrary chosen and used for the synthesis of both RLuc versions. The newly synthesized RLuc variants were placed in the pSS177 vector replacing the standard RLuc. For normalization purposes this plasmids were co-transfected together with pJA24.

### Cell culture

Kc167 cells were grown at 25 C in Schneider’s medium containing 10%FBS (Biochrom) and 1X Penicillin/Streptomycin. S2 cells were grown at 25 C in Express-Five serum-free medium (Life Technologies) supplemented with 2X glutamine and 1X Penicillin/Streptomycin.

### Transfection and luciferase reporter assays

For reporter transfection experiments 0.2 × 10^6^ cells/well were seeded in 96 well plates. Transfections were performed immediately after seeding using Effectene (Qiagen) following manufacturer’s instructions. 20 hours after transfection, cells were lysed and luciferase activity was measured using the dual luciferase assay kit (Promega). In every experiment RLuc activity was normalized against FLuc activity.

DTT (5 mM) or CHX (0.25 ug/ul) were added to the cells 6 h after transfection and allowed to act for 12 h before luciferase measurement.

### Synthesis of RNA reporters

Capped RNA reporters were synthesized using the Megascript T7 kit (Invitrogen). The RLuc and FLuc reporters were amplified by PCR to introduce a 72 nt long polyA at the end of the transcript using a poly(d)T-tailed reverse primer. PCR product were gel purified and used as templates for the *in vitro* transcription reactions in the presence of cap as described previously^12^. All the *in vitro* transcribed reporters contain the CG43674 5′UTR.

### Translation efficiency measurements

For translation efficiency experiments, RLuc and FLuc reporters were *in vitro* transcribed, capped and poly-adenylated as described above, and co-transfected together into S2 cells using the TransMessenger transfection reagent kit (Quiagen). Sixteen hours after transfection each well was divided in two and samples were then processed for Luciferase activity measurement and for RNA extraction followed by Q-RT-PCR to quantify mRNA levels. For calculating translation efficiency, the RLuc/FLuc activity ratio obtained for each of the transfected constructs was divided by the RLuc/FLuc mRNA levels ratio.

### RNAi treatments

For RNAi treatments S2 or Kc167 cells were seeded at a concentration of 85.000 cells/well and treated with 12 ug/mL of dsRNA in 96 well plates. Full sequences of dsRNAs are in Supplementary Table S6. Knock down was allowed to proceed for 5 days. On day 4, cells were transfected with reporters using Efecteene (Qiagen). 20 hours after transection luciferase activity was determined.

### Codon Optimality Measurements

The Renilla Luciferase Codon Optimality Measurement reporters (Fig. 3A), which also contain a Firefly luciferase normalization control, were transfected into Kc167 cells. 20 hours after transfection, cells were lysed and luciferase activity was measured. For calculating the COM for every codon, the RLuc/Fluc ratio was first calculated. The COM of every codon was then normalized by dividing it by the COM value for the best codon for that amino acid. In this way, the COM for the best codon for any given amino acid is 1.

### Translational efficiency and codon optimality

Calculations of the tRNA adaptation index, codon usage and translational efficiency index were performed according to Pechmann and Frydmann 2014.

All indexes were rescaled to 1 by dividing the index of every codon by the maximum index obtained within each amino acid.

### Calculation of Kozak quality

The strengths for all Kozak sequences containing a particular nucleotide at a particular position were collected (e.g. all sequences with an A at position −3), and the mean(pos, nt) and standard deviation(pos, nt) for this distribution was calculated, yielding the data in Supplementary Table S2. The Kozak strength was then calculated as a weighted average of the means for each nucleotide at each position, weighted by the inverse of the standard deviations:

1 $$\frac{{\sum }_{pos=-4}^{+4}{a}_{pos}\bullet mean(pos,nt)\bullet \,1/std\,dev(pos,nt)}{{\sum }_{pos=-4}^{+4}{a}_{pos}\bullet \,1/std\,dev(pos,nt)}$$

where a_pos_ is an empirically derived position-dependent weighting factor that was derived by fitting the model to the measured kozak strengths for the first 264 kozaks that were generated. The weighting factors for positions −4, −3, −2, −1 and +4 were 2.5, 1.4, 3.4, 1.9 and 1 respectively. The correlation between calculated and measured Kozak strengths for all other 427 Kozaks sequences not in the training set is 0.85.

### Data Availability

All data generated or analysed during this study are included in this published article (and its Supplementary Information files).

## Electronic supplementary material

Supplementary Information

Table S1

Table S2

Table S3

Table S4

Table S5

Table S6
