# Supplementary material for: Changes in global translation elongation or initiation rates shape the proteome via the Kozak sequence
Source: Sci Rep. 2018 Mar 5;8:4018. doi: 10.1038/s41598-018-22330-9 (PMC5838203; doi:10.1038/s41598-018-22330-9)

SUPPLEMENTARY INFORMATION

Changes in global translation elongation or initiation rates shape the proteome via the Kozak sequence

Julieta M. Acevedo, Bernhard Hoermann, Tilo Schlimbach and Aurelio A. Teleman

CONTENTS

1. Supplementary Figure Legends .... p. 2

2. Supplementary Table Legends .... p. 5

3. Supplementary Figures .... p. 7

SUPPLEMENTARY FIGURE LEGENDS

Supplementary Figure S1: Mutation of the Kozak sequence on the RLuc reporter affects its translation efficiency.

(A-A') Mutating the Kozak sequence of an RLuc reporter from the 'strong' sequence CACCatgA to the 'weak' sequence TTTTatgA affects both the luciferase activity of the reporter (A), and the translation efficiency of the reporter (A'). The translation efficiency of the reporter, which normalizes out effects on mRNA stability, is calculated as luciferase activity normalized to mRNA levels, measured by Q-RT-PCR. For each panel n=3 biological replicates, error bars: std. dev.

Supplementary Figure S2: Genome-wide characteristics on Drosophila Kozak sequences

(A) The modeled Kozak quality correlates well with measured Kozak strengths. Based on the average and standard deviation of kozak strengths for all sequences with individual nucleotides at a given position (Supplementary Table 2), a mathematical model of Kozak strength was built as described in Materials and Methods. The model was trained with the first 264 Kozak sequences that were generated and measured (red) and an overall correlation between predicted and measured Kozak strength of 0.86 is obtained over the entire set of 680 measured Kozak sequences (blue).

(B) Strength of a Kozak sequence and usage of the Kozak sequence in the fly genome do not correlate very well. Shown here is Kozak strength (x-axis)

versus usage in the Drosophila genome (y-axis) for all Kozak sequences. There is a general tendency for strong Kozak sequences to be more abundant at translation start sites in the fly transcriptome. That said, many strong Kozak sequences are not frequently used, whereas some weak Kozak sequences are present multiple times.

(C) Gene Ontology analysis on weak-Kozak containing transcripts identifies an enrichment for functional groups involved in neuron biology and transcriptional regulation. Enrichment analysis was performed using DAVID v6.7 [14] on the 688 transcripts with Kozak qualities below 70% of the strong Kozak (CACCatgA). GO categories are shown here sorted by Benjamini-corrected p values. Main Figure 1E shows GO terms sorted for fold-enrichment.

Supplementary Figure S3: Targeted RNAi screen against selected translation initiation factors identifies eIF5A as a factor that differentially affects translation of transcripts bearing strong or weak Kozak sequences.

(A) Schematic diagram of the assay setup. Test Renilla Luciferase (RLuc) reporters carrying either a strong or a weak Kozak sequence were co-transfected together with a Firefly Luciferase (FLuc) normalization control carrying a strong Kozak sequence. Graphs in panels B and B' show the RLuc/FLuc ratio, which normalizes out global changes in translation rates as well as technical artefacts, scaled so that the GFP dsRNA values are 1.

(B-B") Targeted RNAi screen against selected translation initiation factors (eIFs) identifies eIF5A as a factor that differentially affects translation of transcripts bearing strong or weak Kozak sequences. S2 (B) or Kc167 (B') cells treated with dsRNA targeting GFP (negative control) or various eIFs were transfected with either a strong Kozak tandem reporter (CACCATGA RLuc-FLuc) or a weak Kozak tandem reporter (TTTTATGA RLuc-FLuc). RLuc/Fluc ratios for each reporter were normalized to GFP dsRNA controls. (B'') Summary of eIFs that differentially affect translation of the strong vs weak Kozak reporter in S2 and Kc167 cells identifies eIF5A as a common hit. (n=4 biological replicates, error bars: std. dev., *statistically significant by t-test followed by Benjamini-Hochberg correction for multiple testing with a false discovery rate of 10%.

(C) Raw luciferase counts for eIF5A knockdown in S2 cells shown in panel B. The raw luciferase counts shown here should be interpreted cautiously, because they are affected by technical issues such as transfection efficiency, amount of plasmid and cells transfected, pipetting, etc. Nonetheless, eIF5A knockdown generally causes a drop in translation rates of both the RLuc test reporters and the FLuc normalization control reporter. The RLuc bearing a strong Kozak sequence is more dramatically inhibited upon eIF5A knockdown compared to the RLuc reporter with a weak Kozak sequence.

(D) 0.25 µg/ml cycloheximide is sufficient to reduce translation. Raw luciferase counts for RLuc in response to a titration of cycloheximide (CHX). The raw luciferase counts shown here should be interpreted cautiously, because they are affected by technical issues such as transfection efficiency, amount of plasmid and cells transfected, pipetting, etc.

Supplementary Figure S4: Translation efficiencies for various RLuc reporters.

(A) The codon quality of a reporter affects its translation efficiency. The translation efficiency of RLuc RNA reporters containing all ‘best’ or all ‘worst’ codons, as measured in this manuscript, was quantified by measuring both luciferase activity and mRNA levels by Q-RT-PCR and normalizing to each other.

(B) The difference in expression observed when comparing RLuc reporters containing a stretch of the best or the worst proline codons after the ATG (see Figure 3A) is due to differences in translation efficiency.

Error bars: std. dev. n=3 biological references.

Supplementary Figure S5: Translation impact of the Kozak sequence strength depends on relative initiation and elongation rates on a transcript.

(A) Schematic representation showing the interplay between the elongation context and the Kozak sequence on the determination of translation efficiency.

SUPPLEMENTARY TABLE LEGENDS

Supplementary Table S1: Results of the Kozak quality screen.

Supplementary Table S2: Average Kozak strength and the standard deviation for all sequences having a given nucleotide at a certain position.

Supplementary Table S3: Predicted Kozak strength from the modeling for all possible Kozak sequences.

Supplementary Table S4: Predicted Kozak strength from the modeling for all *Drosophila* transcripts.

Supplementary Table S5: Codon optimality measurements.

Supplementary Table S6: Full sequences of dsRNAs used in this paper.

Supplementary Figure 1

A   A’

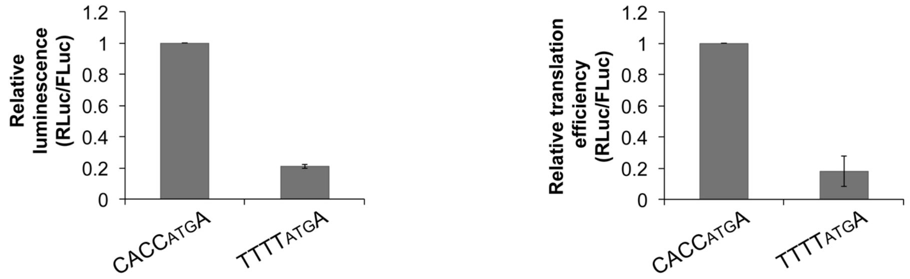

Supplementary Figure 2

A

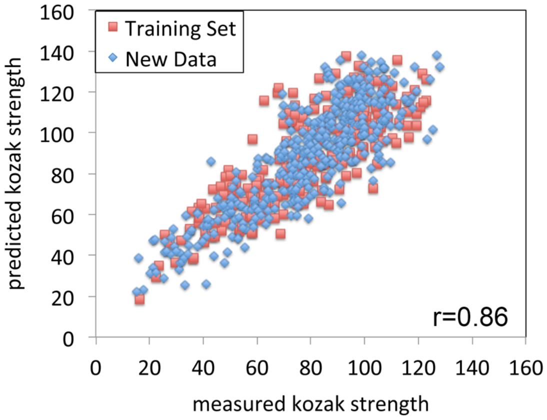

B

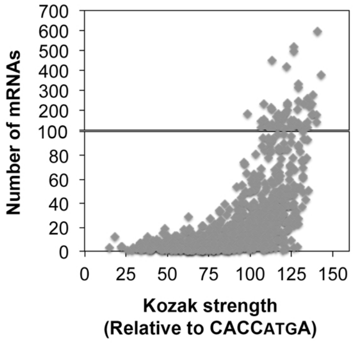

C

Transcripts with weak Kozak sequences

**GO Term** (sorted by p-value)	**Fold Enrichment**	**p value Benjamini**
GO:0030528~transcription regulator activity	1.9	0.004
GO:0003677~DNA binding	1.7	0.006
GO:0032989~cellular component morphogenesis	2.0	0.008
GO:0003702~RNA polymerase II transcription factor activity	2.4	0.008
GO:0003700~transcription factor activity	2.1	0.009
GO:0007423~sensory organ development	2.2	0.009
GO:0003704~specific RNA polymerase II transcription factor activity	3.9	0.011
GO:0030182~neuron differentiation	2.1	0.013
GO:0051252~regulation of RNA metabolic process	1.9	0.013

Supplementary Figure 3

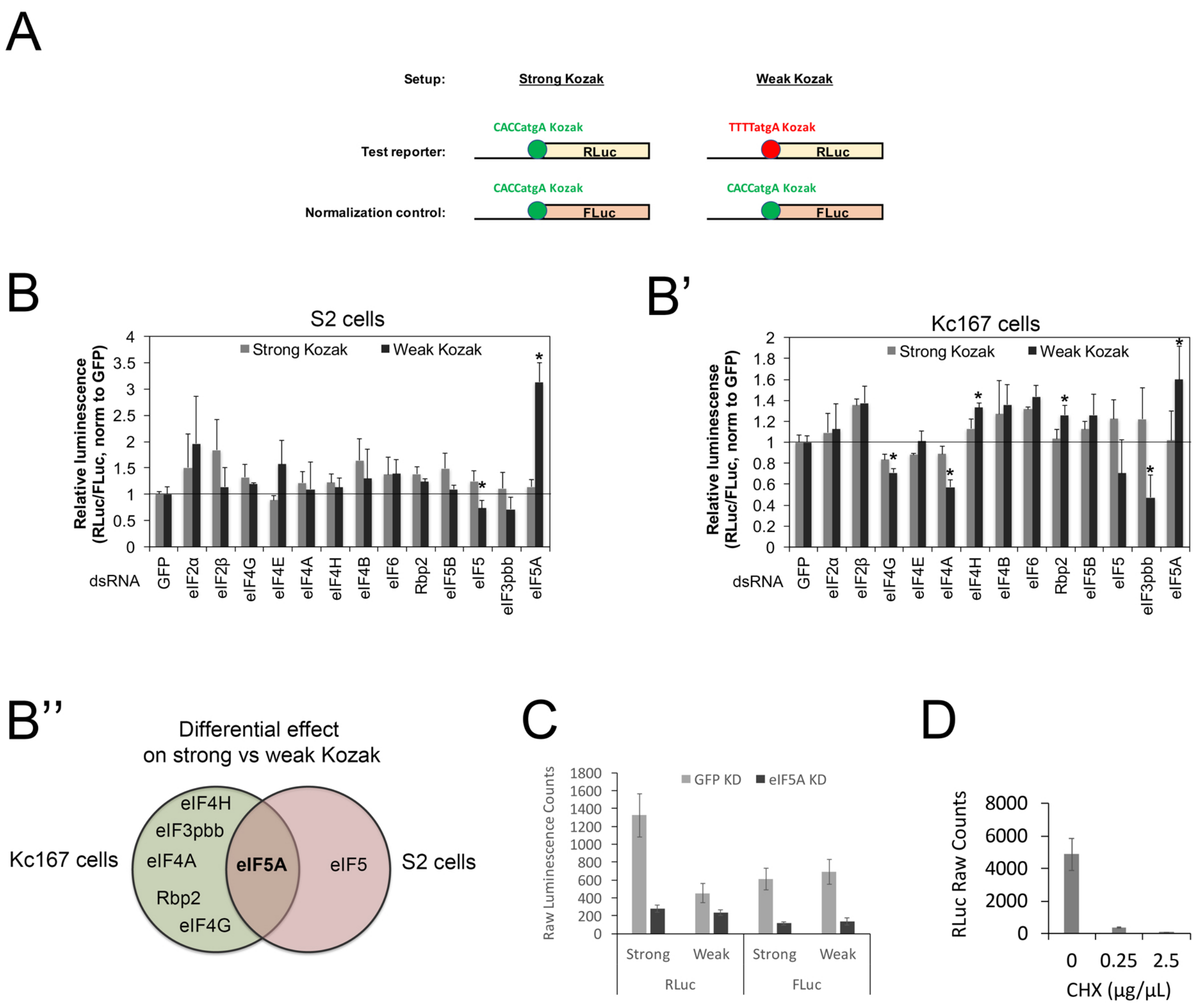

Supplementary Figure 4

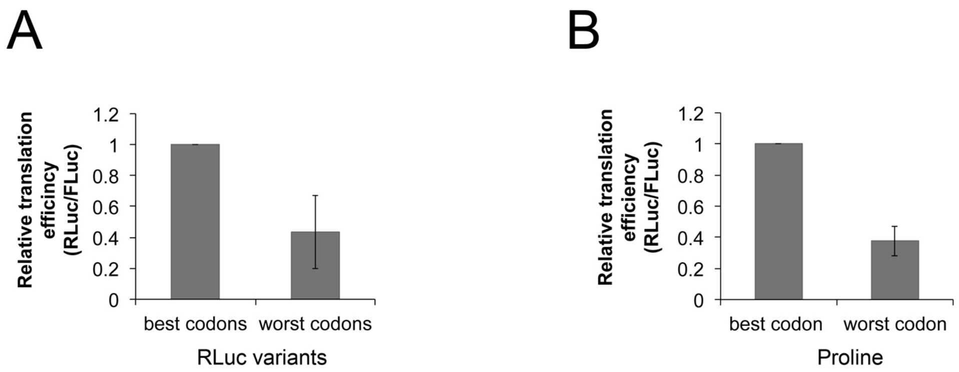

Supplementary Figure 5

A

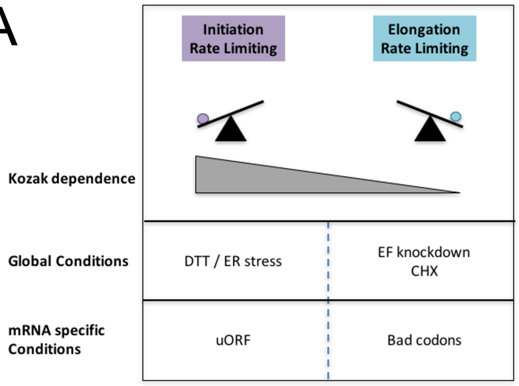

Supplement: Supplementary file 1 — Supplementary Information [file 41598_2018_22330_MOESM1_ESM.pdf]
